# Dr. James A. Libbey

**Published:** 1920-06

**Authors:** 


### OBITUARY

Dr. James A. Libbey. Dr. Libbey, a very prominent member of the profession, died at his home in Swissvale, Pa., near Pittsburg, March 15, 1920, in his seventy-fourth year. Dr. Libbey was a fine gentleman and a professional man of unusual technical capacity. He gave generously of his professional energy and was greatly esteemed by all who knew of his great worth. He gave much for his profession and was greatly beloved by his friends and neighbors.

